# A combination of right ventricular hypertrabeculation/noncompaction and arrhythmogenic right ventricular cardiomyopathy: a syndrome?

**DOI:** 10.1186/1476-7120-6-63

**Published:** 2008-12-23

**Authors:** Ze-Zhou Song

**Affiliations:** 1 Department of Ultrasound, The First Affiliated Hospital, College of Medicine, Zhejiang University, Hangzhou, PR China

## Abstract

A combination of ARVC and RV NVM/HVM, which is extremely rare, to our knowledge, is never reported. RV NVM/HVM could be the cause and consequence of ARVC, or RV NVM/HVM and ARVC could be a consequence of a certain undetermined cause. It must be kept in mind, however, that the interaction of NVM/HVM and ARVC could be in part of pathophysiology mechanism of the combination even if as a consequence of an underlying genetic factor.

## Introduction

Arrhythmogenic right ventricular cardiomyopathy (ARVC) is characterised by fibrofatty replacement of right ventricular myocardium and represents an underdiagnosed cardiac entity leading to sudden cardiac death, syncopes, recurrent ventricular tachycardias and, in some cases, heart failure in a younger population [1].

Noncompaction/hypertrabeculation of ventricular myocardium (NVM/HVM) occurs because of a disorder of endomyocardial morphogenesis that results in a failure of trabecular compaction of the developing myocardium [2]. In adult patients one or more segments of the left ventricle, and sometimes both ventricles, are characterized by numerous sinusoids or trabeculae that are excessive in number and abnormal in prominence and by deep intratrabecular recesses covered by endothelium that exhibits continuity with ventricular endocardium. The prompt recognition of the disease is extremely important because of its high mortality and morbidity due to progressive heart failure, thromboembolism, and malignant arrhythmias.

A combination of ARVC and right ventricular (RV) NVM/HVM, however, which is extremely rare, to our knowledge, is never reported. In addition, although several theories have been advanced, the etiology and pathogenesis of ARVC are still obscure and arrhythmias could be common in patients with ventricular noncompaction. The interesting question, therefore, whether RV NVM/HVM could be the cause and consequence of ARVC, or RV NVM/HVM and ARVC could be a consequence of a certain undetermined cause, is raised. We present a case of a combination of RV NVM/HVM and ARVC in a young man, which was confirmed by echocardiography, magnetic resonance imaging, electrocardiogram and clinical examinations, and approach the possible association of RV NVM/HVM and ARVC.

## Case Report

The patient was a 23-year-old man admitted with mild chest distress and palpitation associated with activity. In past, he was never found to have any heart diseases and relevant history of familial heart diseases.

**Figure 1:**
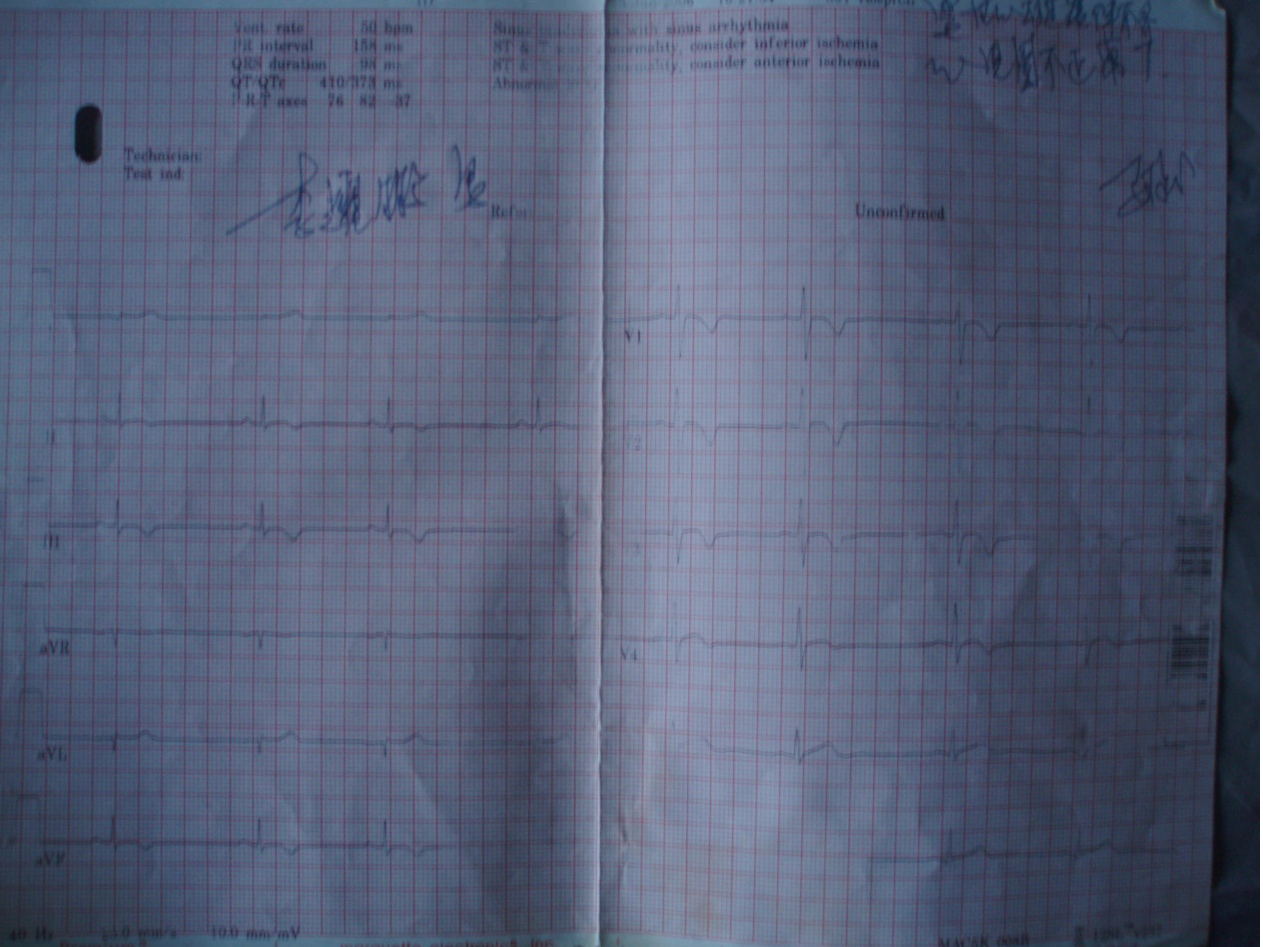
**The 12-lead EKG showing sinus irregularity, right axis deviation, inverted T waves**.

On examination, his blood pressure was 105/75 mm Hg; pulse 76 beat per minute. The EKG and 24-hour Holter monitoring revealed sinus irregularity, right axis deviation, inverted T waves, over 2000 multiple left bundle branch block type extrasystolic ventricular beats and abiogenesis atrial premature beats, but without ischaemic changes on exertion and any evidence of serious ventricular arrhythmias (Fig 1). Chest x-ray revealed the heart to be enlarged, but there was no pulmonary congestion. The laboratory examinations are normal.

**Figure 2:**
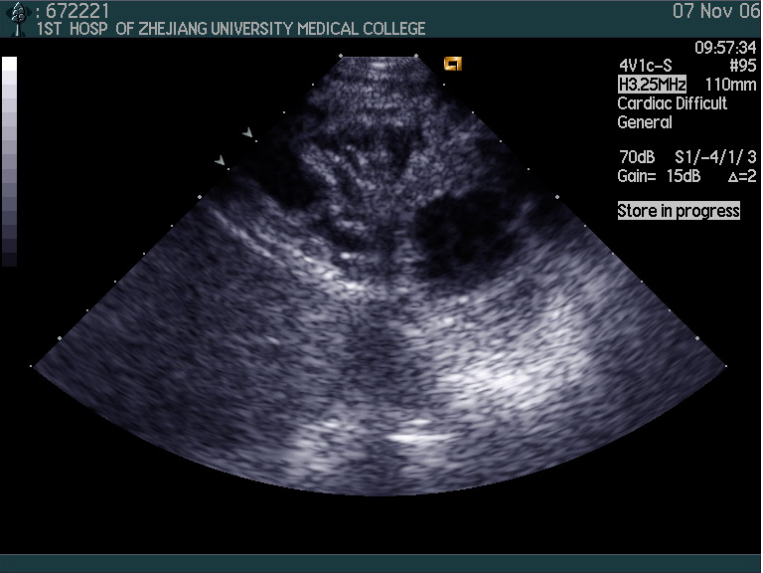
**left ventricular short axis view at next to apex level showing the trabeculations of the right ventricular wall by two-dimension echocardiograms**.

**Figure 3:**
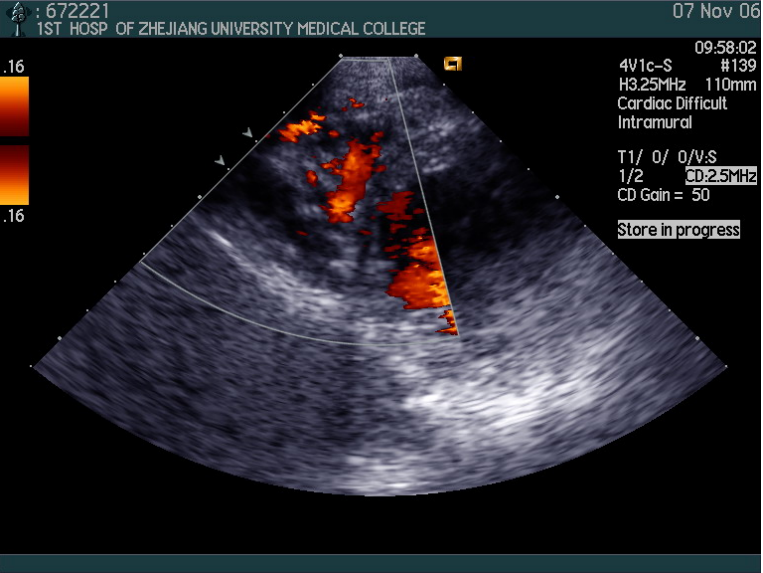
**the same view as figure 2 showing the color flow of deep intertrabecular recesses to right ventricular chamber by color Doppler echocardiograms**.

Transthoracic two-dimensional echocardiography showed prominent trabeculations, with a maximum wall thickness of 41 mm and deep intertrabecular recesses in lateral wall, septum and apex of the right ventricle (Fig 2). The right ventricular and atrium was mildly dilated, lateral wall, septum and apex of right ventricular were hypokinetic with others eukinesia walls and right ventricular function was impaired with an right ventricular area changes fraction of 38%. The left ventricular was not markedly dilated and its function was normal with an left ventricular ejection fraction of 67%. Color and continued wave Doppler echocardiography revealed the color flow of deep intertrabecular recesses to right ventricular chamber (Fig 3) and tricuspid regurgitation with the maximum velocity 2.21 meters per second which confirm the right peak systolic pressure (29.5 mmHg) which is the same as pulmonary arterial systolic pressure is among normal values.

**Figure 4:**
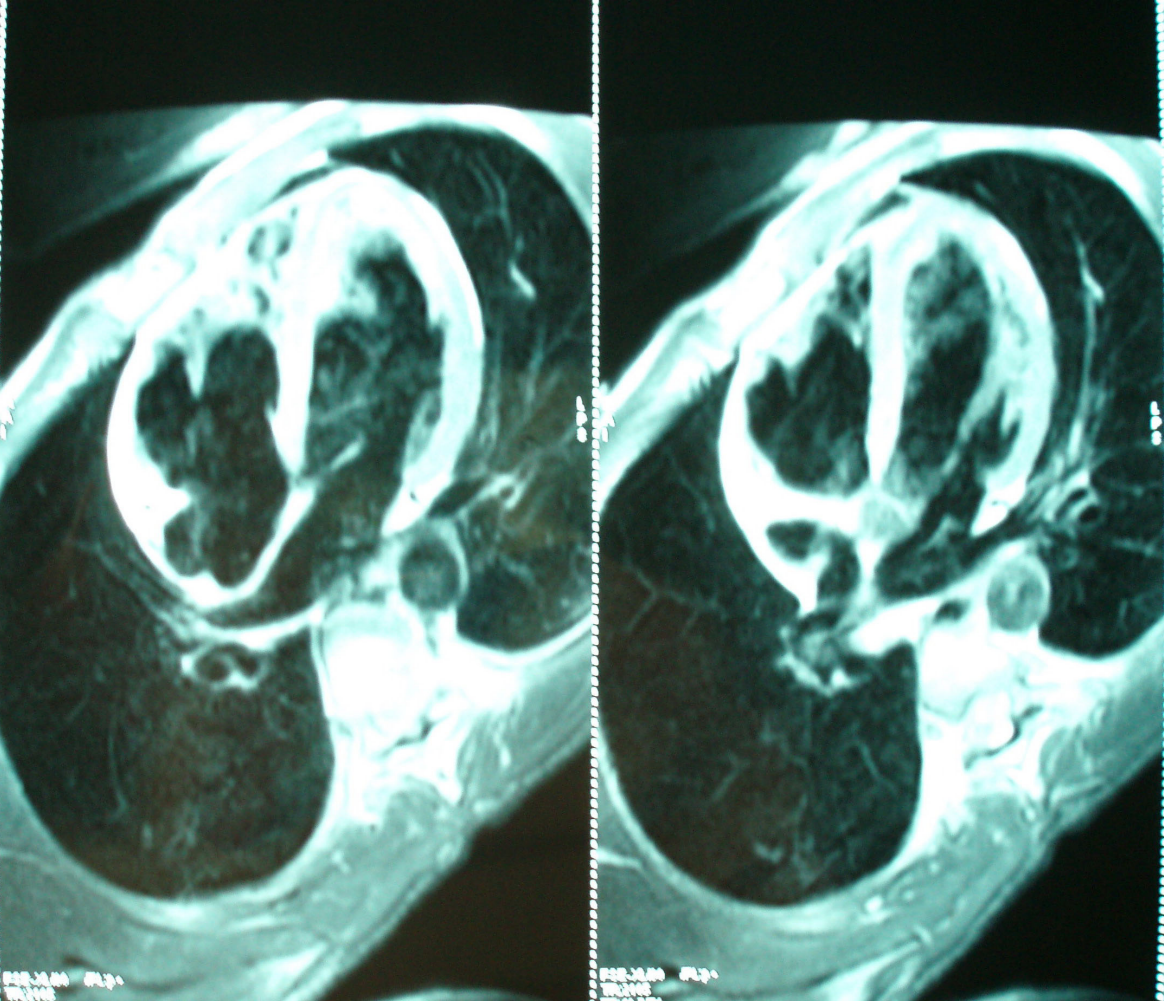
**Four chamber view showing the trabeculations of the right ventricular wall by Magnetic resonance imaging**.

Magnetic resonance imaging showed a double-layered appearance with noncompacted endomyocardial segment and compacted epimyocardial segment in the lateral wall and the apex on the four-chamber view (Fig. 4). There was dyskinetic movement in the right ventricular apex. Marked trabeculations and deep intratrabecular recesses in the anterior, lateral, and inferior segments on the short-axis view were observed. The right ventricle was dilated, and it had marked trabeculations in the apical portions of the right ventricle and dyskinetic movement in the lateral wall, septum and apex of right ventricular segments of the right ventricular.

At presentation, the patient complaint less release of mild chest distress and palpitation associated with activity despite of optimization of anti-arhythmia and anti-heartfailure therapy and refused ICD or CRT.

## Discussion

Although there is no established criteria for noncompaction in the right ventricle, RV NVM/HVM has been reported in some cases in whom MRI was used in addition to echocardiography [3-5]. Echocardiographic quantitation of right ventricular function has been elusive because of the asymmetric shape of the ventricle, which does not fit well with geometric assumptions and becomes even less predictable as dilatation and hypertrophy occur [6]. Although recent computational methods with lamb heart models and two-dimensional echocardiography have shown promise in an in vitro test [7], this has not yet been clinically proven as applicable. Because morphological assessment of the right ventricle is often difficult in adult patients and because the echocardiographic image quality is operator dependent, the prevalence of RV NVM/HVM may have been underestimated in the past. MRI, however, may accurately depict morphological abnormalities such as prominent trabeculations with deep intratrabecular recesses and motion abnormalities of RV as the present case and, therefore, strengthen the diagnosis of RV NVM/HVM in the present case.

To the best of our knowledge no case of a combination of RV NVM/HVM and ARVC has been reported in the literature. And then, what on earth is the possible association of RV NVM/HVM and ARVC?

The possible mechanism for arrhythmogenesis in NVM/HVM might be a result of abnormalities in the cardiac conduction system. This assumption is substantiated by the finding of Purkinje's fibers as components of false tendons, which are found frequently in NVM/HVM [8]. That is to say, RV NVM/HVM may be the cause of ARVC in the present case. NVM/HVM might be the result of an adaptation to special hemodynamic conditions. These theories are supported by the finding that a trabeculated myocardium has a markedly different viscoelastic behavior, influencing the rate and magnitude of contraction and relaxation, than the compact myocardium [9] the icefish heart function as a specialized volume pump that moves large stroke volumes at a low heart rate, but is not able to produce high pressures [10]. Also in human beings, the right ventricle, which belongs to a low pressure system, is more trabeculated than the left ventricle. ARVC, however, is one of the few myocardial diseases that cause RV heart failure without pulmonary hypertension as the present case. The mechanism for the RV failure is dilation, thinning of the wall and progressive loss of contractile function because of myocardial atrophy. NVM/HVM, therefore, might be the result of an adaptation to RV failure induced by ARVC. That is to say, RV NVM/HVM may be the consequence of ARVC in the present case. The so-called ARVC is a primary heart muscle disorder characterized by a progressive loss of myocardium, with a peculiar fatty or fibrofatty replacement, that accounts for the onset of cardiac electrical instability [11]. The finding of a gene defect localized on chromosome 14q23-q24 favors a genetically determined atrophy, as observed in the skeletal muscle of patients with Duchenne's and Becker's diseases, and the term "myocardial dystrophy" might appear to be the most appropriate. On the other hand, although NVM/HVM is usually regarded as a congenital disorder, in single patients with Duchenne muscular dystrophy [12], or Becker muscular dystrophy [13], metabolic myopathy [14], NVM/HVM developed during their lifetime, which were termed acquired NVM/HVM by Finsterer and Bleyl et al [12-14]. That is to say, a combination of RV NVM/HVM and ARVC may be a consequence of an underlying genetic factor.

## Competing interests

The author declares that they have no competing interests.

## Consent

Written informed consent was obtained from the patient for publication of this case report and accompanying images. A copy of the written consent is available for review by the Editor-in-Chief of this journal.
